# Tumour Angiogenesis in Uveal Melanoma Is Related to Genetic Evolution

**DOI:** 10.3390/cancers11070979

**Published:** 2019-07-13

**Authors:** Niels J. Brouwer, Gülçin Gezgin, Annemijn P.A. Wierenga, Inge H.G. Bronkhorst, Marina Marinkovic, Gregorius P.M. Luyten, Mieke Versluis, Wilma G.M. Kroes, Pieter A. van der Velden, Robert M. Verdijk, Martine J. Jager

**Affiliations:** 1 Department of Ophthalmology, Leiden University Medical Center, Albinusdreef 2, 2333 ZA Leiden, The Netherlands; 2 Department of Ophthalmology, Jeroen Bosch Hospital, 5223 GZ ‘s-Hertogenbosch, The Netherlands; 3 Department of Clinical Genetics, Leiden University Medical Center, 2333 ZA Leiden, The Netherlands; 4 Department of Pathology, Leiden University Medical Center, 2333 ZA Leiden, The Netherlands; 5 Department of Pathology, Erasmus University Medical Center, 3015 GD Rotterdam, The Netherlands

**Keywords:** uveal melanoma, angiogenesis, oncology, BAP1, VEGF-B, chromosomes, macrophages

## Abstract

Increased angiogenesis is associated with a higher metastasis- and mortality rate in uveal melanoma (UM). Recently, it was demonstrated that genetic events, such as 8q-gain and BAP1-loss, influence the level of immune infiltrate. We aimed to determine whether genetic events, and specific cytokines, relate to angiogenesis in UM. Data from UM patients who underwent enucleation between 1999 and 2008 were analysed. Microvascular density (MVD) and the presence of infiltrating immune cells were determined with immunohistochemistry (IHC) and immunofluorescence in 43 cases. Chromosome status, BAP1 IHC and mRNA expression of angiogenesis-related genes were known in 54 cases. Tumours with monosomy 3/BAP1-loss showed a higher MVD compared to tumours with disomy 3/normal BAP1 expression (*p* = 0.008 and *p* = 0.004, respectively). Within BAP1-positive lesions (*n* = 20), 8q-gain did not relate to MVD (*p* = 0.51). A high MVD was associated with an increased expression of angiopoietin 2 (ANGPT2) (*p* = 0.041), Von Willebrand Factor (VWF) (*p* = 0.010), a decreased expression of vascular endothelial growth factor B (VEGF-B) (*p* = 0.024), and increased numbers of tumour-infiltrating macrophages (CD68+, *p* = 0.017; CD68+CD163+, *p* = 0.031) and lymphocytes (CD4+, *p* = 0.027). Concluding, vascular density of UM relates to its genetic profile: Monosomy 3 and BAP1-loss are associated with an increased MVD, while an early event (gain of 8q) is not independently related to MVD, but may initiate a preparation phase towards development of vessels. Interestingly, VEGF-B expression is decreased in UM with a high MVD.

## 1. Introduction

Uveal melanoma (UM) is the most common ocular malignancy in Caucasian adults. The disease is often lethal with up to 50% of patients developing metastases [1]. In recent years, research has focussed on targeted and immunotherapeutic therapies, as promising results were obtained in the treatment of, for example, cutaneous melanoma. Results in UM are disappointing; however, and questions remain regarding the mechanisms leading to metastases and the tumour’s resistance to treatment. We sought to learn more about the relation between the tumour’s immunological microenvironment and the development of angiogenesis, which is an important parameter in growth and behaviour of UM, and a potential target for therapy.

Restricted by the limits of diffusion (1–2 mm), an expanding UM requires new vessels to grow. The concept of the “angiogenic switch” describes the turning point between an initial phase of slow, avascular growth into a phase with more rapid growth and angiogenesis. Angiogenesis can be studied using micro-vascular density (MVD). An increased MVD has been associated with a higher metastasis rate [2] and mortality rate in UM [3,4]. Since UM metastasizes solely via the haematogenous route, it is logical to assume a relation between growth of intra-tumoural vessels and systemic spread. This concept led to the hypothesis that anti-angiogenic therapy could be used to treat UM or its metastases. One recent study reports a potential benefit of treatment with anti- vascular endothelial growth factor (VEGF) therapy in metastatic UM [5], but others stress that anti-angiogenic therapy has been unsuccessful in UM [6]. This illustrates that there are still questions to be answered to fully understand angiogenesis in UM. Scientific interest in tumour vascularization has recently increased as new targets, including hypoxia signalling [7], have proven to be promising in the therapeutic approach of UM [8].

While angiogenesis describes the formation of endothelial-lined new vessels, it is important to note that other mechanisms resulting in intra-tumoural vascular channels have been recognized in UM [9,10]. This phenomenon is called “vascular mimicry”, and can be depicted by periodic acid–Schiff (PAS) staining of extravascular matrix patterns. The presence of so-called loops and networks in UM was related to the MVD [11] and worse prognosis [11]. However, the exact function and development of these channels remains debated.

The MVD of UM is known to be associated with the tumour’s immune infiltrate. Studies on this topic have focussed mainly on the relation with an increased presence of macrophages, describing their pro-angiogenic properties. An association between a higher MVD and increased numbers of all macrophages (CD68+ cells) [12] and M2 type macrophages (CD68+CD163+ cells) [2] has been established. Recently, it was found that genetic changes that reflect the evolution of UM relate to the type of immune infiltrate in tumour samples [13]. Gain of chromosome 8q (an early event in UM development [14,15]) is related to an increased presence of macrophages, while the loss of BAP1 expression (a later event) is related to an increased presence of T cells. Currently, the roles of 8q gain or BAP1 loss in angiogenesis are unknown. As monosomy 3 and BAP1 loss are very important for prognosis in this disease [16,17], and they play a role in developing an inflammatory phenotype [18], we wondered if angiogenesis as demonstrated by MVD is similarly regulated by these genetic events.

We hypothesize that genetic changes not only influence the immunological microenvironment, but also drive angiogenesis in UM, and that MVD is a consequence of a highly-malignant genetic profile. We set out to test this, and also analysed if several angiogenesis-related cytokines are expressed in relation to the development of tumour vascularity of primary UM.

## 2. Results

### 2.1. A High MVD Relates to a Mixed/Epithelioid Cell Type and a Worse Clinical Outcome

As a high MVD is known to be associated with a bad prognosis in UM, we first determined whether our population confirmed the existing assumptions. The MVD had been determined by counting CD34-expressing vessels in sections of 43 UM, using a well-established technique as presented by Makitie et al. [3]. The mean age at enucleation of these patients was 60.6 years. The median largest basal diameter (LBD) of the tumours was 13.0 mm. Following the American Joint Committee on Cancer (AJCC) tumour-node-metastasis (TNM) staging criteria, three (7%) tumours were Stage T1, 22 (51%) Stage T2 and 18 (42%) Stage T3. Further details on the patient and tumour characteristics are provided in Table 1.

**Figure 1:**
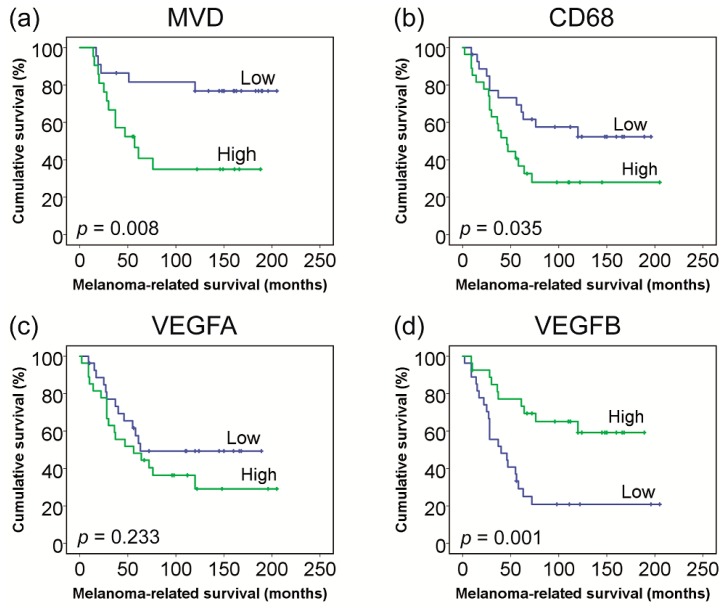
Patient survival in relation to MVD and mRNA gene expression. Groups (high vs. low) were based on the median vessel counts and mRNA gene expression values. (**a**) **Immunohistochemistry** (IHC) counts of MVD (*n* = 43), (**b**) mRNA gene expression of CD68 macrophages (*n* = 54), (**c**) mRNA gene expression of VEGF-A (*n* = 54), and (**d**) mRNA gene expression of VEGF-B (*n* = 54).

The median MVD count was 89.0 CD34+ vessels/mm^2^ (Range: 28.0–202.0). A high MVD was related to a mixed/epithelioid cell type (*p* = 0.004), but not to gender (*p* = 0.89), age (*p* = 0.25), tumour stage (*p* = 0.23) or tumour pigmentation (*p* = 0.45). When looking at a comparison with vascular mimicry, the median MVD count increased from cases without loops or networks (71.0 vessels/mm^2^), to those with loops only (83.0 vessels/mm^2^) and those with both loops and networks (102.0 vessels/mm^2^) (*p* = 0.052). The median follow-up time was 120 months (range: 14–205 months). In total, 18 patients (42%) developed metastasis and died of melanoma-related causes. A high MVD was related to the development of metastasis (*p* = 0.009) and the occurrence of metastasis-related death (*p* = 0.009) (Table 1), similarly presented in Figure 1a.

### 2.2. MVD Relates to the Expression of Several Angiogenesis-Related Genes

To identify the relevance of angiogenesis-related pathways in UM, we related the MVD to the mRNA expression levels of several selected angiogenesis-related genes. Data on MVD as well as mRNA expression was available for 28 UM patients. Potentially-relevant genes were selected from the literature because of their theoretical role in angiogenesis, such as those coding for VEGF-A/B/C, HIF1a, ANGPT1/2, and PDGF-A. We also analysed vessel markers such as CD34 and PECAM1 (CD31). Patient and tumour characteristics of the 28 patients are provided in Table S1.

A high MVD (defined as number of CD34+ vessels/mm^2^) was correlated with an increased mRNA expression of the vessel markers CD34 (*p* = 0.007) and PECAM1 (*p* = 0.055), the pro-angiogenic factors ANGPT2 (*p* = 0.041) and VWF (*p* = 0.010), and a decreased expression of VEGF-B (*p* = 0.024). The expression of VEGF-A was not related to MVD (*p* = 0.98), while the expression of HIF1a (*p* = 0.089) and CDH1 (*p* = 0.079) demonstrated a trend towards an increase with a higher MVD, but this did not reach statistical significance (Table 2). A low expression of VEGF-B (but not VEGF-A) was related to worse survival in a Kaplan–Meier analysis (Figure 1c,d).

### 2.3. MVD Relates to Increased Numbers of Macrophages (CD68+) as Well as T Cells (CD4+)

Previously, MVD was found to correlate with the number of tumour-infiltrating macrophages [2]. This was confirmed in the current set of 43 tumours, by determining the numbers of lymphocytes (CD3+, CD4+, CD8+, FoxP3+; using immunofluorescence (IF) [19]) and macrophages (CD68+, CD163+, CD68+CD163+; using IF [2]). A higher MVD was significantly associated with an increased number of CD68+ (r 0.361, *p* = 0.017), and CD68+CD163+ (r 0.329, *p* = 0.031) macrophages, and also with the number of CD4+ (r 0.336, *p* = 0.027) T cells. A trend was observed between a high MVD and increased counts of CD3+ (r 0.287, *p* = 0.062), CD8+ (r 0.271, *p* = 0.062) and FoxP3+ (r 0.283, *p* = 0.078) cells.

### 2.4. MVD Relates to Monosomy 3 and BAP1 Loss, but Not to Gain of Chromosome 8q

**Figure 2:**
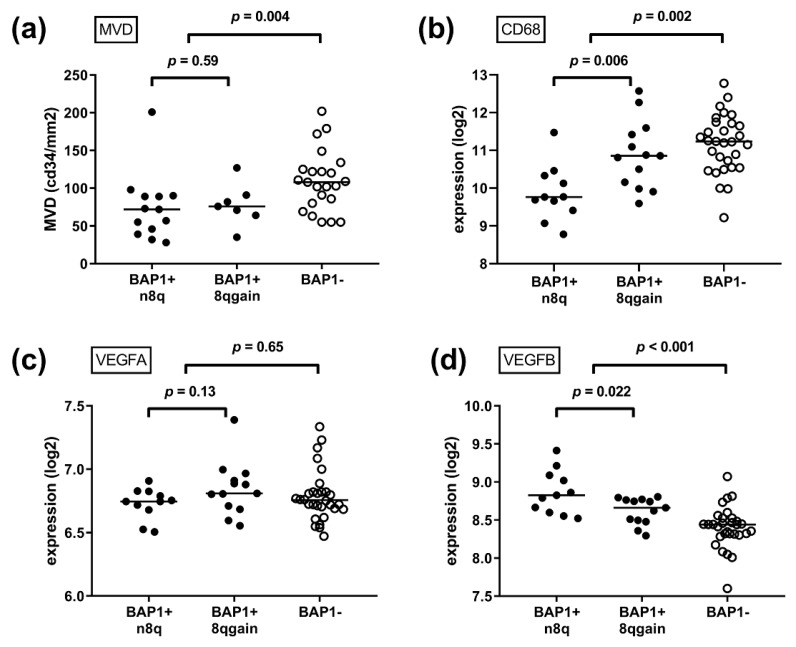

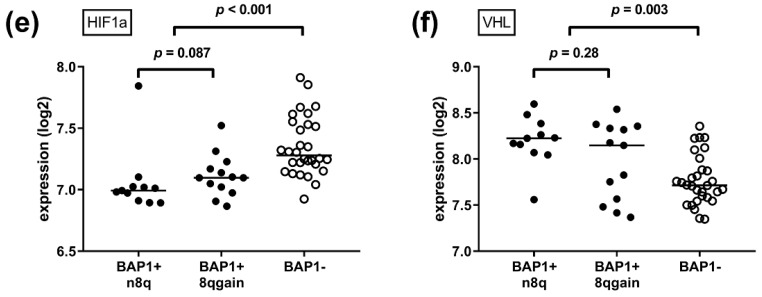
Tumour genetics in relation to MVD and mRNA gene expression. Gain of chromosome 8q is an early event in UM development, while loss of BAP1 is a later event. (**a**) IHC counts of MVD (*n* = 43), (**b**) mRNA gene expression of CD68 macrophages (*n* = 54), (**c**) mRNA gene expression of VEGF-A (*n* = 54), (**d**) mRNA gene expression of VEGF-B (*n* = 54), (**e**) mRNA gene expression of HIF1a (*n* = 54), and (**f**) mRNA gene expression of VHL (*n* = 54). (*p*-values were obtained using Mann–Whitney *U* tests, comparing BAP1+ and n8q with BAP1+ and 8qgain patients, and all BAP1+ with all BAP1− patients. Abbreviations: BAP1+, BAP1-positive; BAP1−, BAP1-negative; n8q, normal chromosome 8q; 8qgain, gain of chromosome 8q).

**Figure 3:**
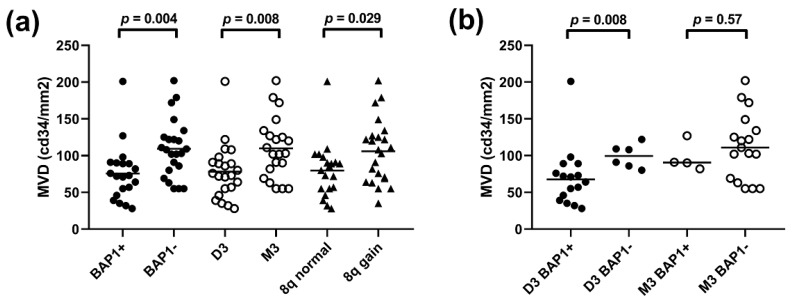
Tumour genetics in relation to MVD. (**a**) Within all 43 patients, patients were compared based on status of BAP1, chromosome 3 or chromosome 8q. (**b**) Within either disomy 3 (*n* = 22) or monosomy 3 (*n* = 21) patients, patients were compared based on status of BAP1. (*p*-values were obtained using Mann–Whitney *U* tests. Abbreviations: BAP1+, BAP1-positive; BAP1−, BAP1-negative; D3, Disomy 3; M3, Monosomy 3; 8q normal, normal chromosome 8q; 8q gain, gain of chromosome 8q).

To investigate the association between tumour genetics and angiogenesis, the status of chromosome 3, chromosome 8q and the expression of the BAP1 protein were determined in 43 patients. Tumours with monosomy 3 (*n* = 21) had a higher MVD compared to tumours with disomy 3 (*n* = 22, *p* = 0.008). Similarly, BAP1-negative tumours (*n* = 23) had a higher MVD compared to BAP1-positive tumours (*n* = 20, *p* = 0.004) (Figure 2a and Figure 3a). To investigate the role of BAP1 independently of chromosome 3 status, we analysed the association between BAP1 and MVD within groups of disomy 3 and monosomy 3 tumours separately. Within the group of disomy 3 tumours (*n* = 22), BAP1 loss (*n* = 6) was still related to a higher MVD compared to tumours that expressed BAP1 (*n* = 16, *p* = 0.008). Within the group of monosomy 3 tumours (*n* = 21), this association could not be established, but that may be due to a small sample size as only four out of 21 tumours with monosomy 3 had not lost their BAP1 expression (Figure 3b). For further comparisons, we focussed on BAP1 expression.

While monosomy 3 (or loss of BAP1) is considered a late event in the development of UM, gain of 8q is an early event [14,15]. When analysing all cases, gain of chromosome 8q was related to an increased MVD (*p* = 0.029) (Figure 3a), but most tumours with gain of 8q also demonstrated BAP1 loss. When we analysed the relationship of 8q gain within tumours that still expressed BAP1 (*n* = 20), 8q gain was not related to MVD (*p* = 0.59) (Figure 2). As only two of the BAP1-negative tumours demonstrated normal 8q, we cannot conclude on the effect of 8q gain within BAP1-negative lesions.

### 2.5. Expression of Angiogenesis-Related Genes Is Related to Genetic Progression of UM

Earlier in this study, we noticed that several angiogenesis-related genes are related to the MVD in UM. We wondered whether the expression of these genes may be related to genetic progression (early 8q gain and later BAP1 loss) in the 54 cases with data on tumour genetics and mRNA gene expression (Table S2). First, we compared all BAP1-positive (*n* = 24) with all BAP1-negative (*n* = 30) lesions. Loss of BAP1 expression was associated with an increased expression of HIF1a and ANGPT2, and a decreased expression of VEGF-B, and VHL (all *p* < 0.05). When looking at vascular markers and infiltrate, BAP1 loss was associated with an increased mRNA expression of vascular markers CDH1, PECAM1, VWF and infiltrate markers CD3, CD4, CD8 and CD68 (Table S3).

We corroborated these findings using the TCGA dataset, and found similar results for the association between BAP1 loss and increased expression of HIF1a, ANGPT2, CDH1, PECAM1, CD3 and CD8, and between BAP1 loss and a decreased expression of VHL and VEGF-B. Interestingly, in the TCGA data, BAP1 loss was also related to an increase of VEGF-A and ANGPT1 (while this was not observed in the Leiden data) (Table S4).

Second, we evaluated the role of chromosome 8q within the BAP1 expressing tumours. Although we identified that gain of 8q is not independently related to MVD, a previous study demonstrated that gain of 8q is related to increased counts of (pro-angiogenic) macrophages [13]. We confirm that within the group of BAP1-expressing tumours from Leiden, gain of 8q was associated with a higher mRNA expression of CD3 (lymphocytes, *p* = 0.026) and especially of more CD68 (macrophages, *p* = 0.007). When examining cytokines, within the group of BAP1-expressing tumours, 8q gain was related to an increased expression of ANGPT2 (*p* = 0.040) and a decreased expression of VEGF-B (*p* = 0.022) and VEGF-C (*p* = 0.026) (Table S3). The relation between 8q gain, BAP1 loss and the expression of several of the investigated genes is presented in Figure 2. During tumour progression, VEGF-B and VHL decrease, while HIF1a increases.

In the TCGA data, gain of 8q was similarly related to an increased expression of CD68 (macrophages), and expression of PDGF-A, but no relation with any of the other cytokines was observed (Table S4).

## 3. Discussion

As we already know that genetic events are closely associated with the immunological microenvironment in UM, including the presence of macrophages and lymphocytes, we analysed whether genetic events also play a role in the MVD and the expression of angiogenic factors in UM. We demonstrate an important association between monosomy 3/BAP1 loss and the expression of several angiogenesis-related genes and MVD. Gain of chromosome 8q was not independently related to MVD, but it was related to a differential expression of several angiogenesis-related genes: The expression of pro-angiogenic ANGPT2 was increased, and (presumably) anti-angiogenic VEGF-B was decreased with 8q gain. This may indicate that 8q gain is involved in a preparation phase towards the development of more vessels. However, it looks as if a true increase in MVD can only be accomplished by a series of events, in which the BAP1 gene may play an important role. This idea may fit well into the concept of the angiogenic switch, describing a slow early avascular tumour growth phase, followed by a more rapid growth with vascular development.

By using mRNA expression techniques, a comprehensive analysis of angiogenesis-related genes was performed. Interestingly, VEGF-A expression was not related to MVD in our data. VEGF-A is the main type of VEGF and is considered to be of importance for the development of new blood vessels. Even more, various studies demonstrated that elevated levels of VEGF-A are present in the aqueous and vitreous of UM eyes [20,21,22]. An explanation for our finding could be that VEGF-A is either important for the most initial development of vasculature, or for the maintenance of previously-developed vasculature, while other factors influence a further increase in MVD.

Our results show that VEGF-B may have a much more important role in angiogenesis in UM than previously thought. An abundant expression of VEGF-B was reported earlier in UM cell lines [23], but the role of VEGF-B has always been described as enigmatic. Interestingly, in our data the expression of VEGF-B correlated negatively with the MVD. A relation between VEGF-B and MVD is unreported in UM, but the function of VEGF-B was recently studied in a murine model, using a cutaneous melanoma cell line. Enforced expression of VEGF-B led to suppressed primary tumour growth in mice and a reduced MVD, but more metastases [24]. It was proposed that an increase in VEGF-B causes increased vascular leakiness, a high degree of hypoxia, with increased numbers of tumour-infiltrating macrophages, leading to a metastasis-promoting environment [24]. Indeed, a relation between high mRNA expression of VEGF-B and worse survival was found in patients with lung squamous cell carcinoma and non-ocular melanoma [24]. Interestingly, the relation between a low VEGF-B expression and metastasis development in our UM data did not follow the positive correlation that was reported with other cancers. In the Leiden data, the development of metastasis was not related to VEGF-A expression, but it related to a decreased expression of VEGF-B. In the TCGA data, both an increased expression of VEGF-A and a decreased expression of VEGF-B were related to more metastasis formation. These observations may indicate that the function of VEGF-B regarding tumour behaviour is different in UM compared to other tumours.

New insights in ischemic signalling pathways have drawn attention to HIF1a-regulated angiogenesis in UM, and new drugs targeting these pathways are being developed [8]. In our study, mRNA expression of HIF1a was not significantly related to an increasing MVD (*p* = 0.089). This may be due to sample size, as Mouriaux demonstrated a link between HIF1a expression and vascular marker CD31 in a larger set of 56 UM [25]. A recent study in UM cell lines on HIF1a-related angiogenesis showed that both VEGF and ANGPTL4 are promotors for tubule formation [26]. The effectors of HIF1a may therefore include multiple pathways, stressing that not only VEGF-A related pathways may have relevance. We furthermore demonstrate that BAP1-loss is strongly related to HIF1a expression, implicating the HIF1a-mediated pathways of angiogenesis in the later steps of UM progression. However, the exact role of BAP1 in UM development, including angiogenesis, is not well understood. It can be hypothesized that BAP1 loss leads to an upregulation of HIF1a via the NF-kB cascade, as BAP1 was shown to suppress this pathway in human oesophageal carcinoma [27], and BAP1 loss was found to be related to an increased NF-kB expression in UM [28], but the mechanism needs to be investigated further.

Previously, associations were reported between the MVD and the presence of tumour-associated macrophages (CD68+ cells [3], and CD68+CD163+ cells [2]). It was hypothesized that macrophages have a pro-angiogenic effect by, for example, secreting VEGF. We confirm the relationship between a high MVD and increased counts of CD68+ cells, and also find an association with CD4+ cells. The numbers of these cells are highly correlated [19]; however, and it has been observed that activated macrophages can attract a T cell infiltrate [29]. It should; therefore, be further studied if T cells have an independent relation to vasculature or whether they act downstream of the presence of macrophages.

Regarding tumour size, we identified that LBD (B = 5.15; 95%CI 0.73 to 9.58; *p* = 0.024), but not tumour prominence (B = −3.63; 95%CI −9.07 to 1.80; *p* = 0.18), was related to MVD in a univariate linear regression analysis. Adjusting for BAP1 status, there was still a trend that LBD related to MVD (B = 4.16; 95%CI −0.10 to 8.42; *p* = 0.055) while BAP1 status related to MVD as well (B = −29.08; 95%CI −53.01 to −5.14; *p* = 0.019). Makitie detected a weak correlation between MVD and increasing LBD as well as with prominence, but had a larger study group of 134 UM, and he did not know the BAP1 status [3]. Our results may imply that tumour size alone (with presumed increased hypoxia) is not the driver of angiogenesis, and that genetics are an important determinant. It would be interesting to investigate whether increased vascularity explains why some large, yet disomy 3/BAP1-positive tumours become metastatic. However, our numbers were not sufficient to examine this relation.

Opposed to the relations we identified between tumour genetics and MVD, the presence of extravascular matrix patterns demonstrated a slightly different relationship. As with MVD, the status of chromosome 8q was not related to the presence of loops (*p* = 0.89) or networks (*p* = 0.32). However, cases with monosomy 3 demonstrated more often loops (*p* = 0.038) and networks (*p* = 0.016). This finding is line with the earlier observation of Onken et al. that the presence of loops and networks relates to gene expression profile class II UM [30]. Interestingly, loops and networks did not relate to BAP1 status (*p* = 0.13 and *p* = 0.13, respectively). This finding may underline the different aetiology of the vascular structures, though we cannot but speculate on the role of BAP1 in this finding.

A limitation of this study was that mainly larger tumours were included as all samples were obtained from enucleated eyes. This also limited the variation in tumour size. It can be expected that new vasculature is especially important for larger lesions; however, so it may be of no major concern that few small-sized tumours were studied.

While the presence of intra-tumoural vessels and infiltrate was analysed with immunohistochemistry (IHC) and IF, respectively, the expression of the various angiogenic factors was analysed using mRNA. This is a well-established technique capable of identifying pathways of interest. However, there may be differences between mRNA gene expression and protein production. Future studies could investigate how our findings, which we corroborated using the mRNA expression data of the TCGA project, relate to data on protein expression of the respective factors.

Our study implicates that angiogenesis should be studied together with the genetic background of UM. An important future project could be to study if anti-angiogenic treatment is more effective in specific (genetic) sub groups of UM. As we show that vascularity is related to genetics, it may be that mainly highly-vascularized lesions are effectively attacked with those treatments or that genetic profiling can predict responses to anti-angiogenic therapy. Another project may be to study which other genes on chromosome 3, besides BAP1, are important for MVD development. In this, it may be important to consider the role of VHL as the VHL gene is, like BAP1, located on chromosome 3. As we demonstrate a role for VEGF-B in UM angiogenesis, the exact role of this cytokine and the relevance for anti-angiogenic therapy should be investigated.

## 4. Materials and Methods

### 4.1. Patient Selection

Tumour samples were obtained from eyes with UM that had been primarily enucleated at the Leiden University Medical Center (LUMC, Leiden, The Netherlands) between 1999 and 2008. Clinical data was retrieved from patient medical files. Survival data was complemented with data from the Dutch national cancer registry (RANK). The study was approved by the Biobank Committee of the LUMC (19.060.CBO/uveamelanoomlab-2019-1), and adhered to the tenets of the Declaration of Helsinki.

The current study includes a previously reported set of 43 tumours with data on MVD, tumour infiltrate and tumour genetics (Table 1) [2,19], and an additional (partially overlapping) set of 54 tumours with data on mRNA gene expression and tumour genetics (Table S2). Of all patients, 28 cases with combined data on MVD and mRNA gene expression were available (Table S1). Clinical data and survival data of all patients were updated until 1 March 2017.

### 4.2. Histopathology

Tumour material was snap frozen using 2-methyl butane and later used for DNA and RNA isolation. Remaining tumour material was fixed in 4% neutral-buffered formalin for 48 h and embedded in paraffin. Haematoxylin/eosin-stained 4 μm sections were reviewed by an ocular pathologist for confirmation of the diagnosis and evaluated for histologic parameters (LBD, prominence, cell type, pigmentation). The eighth edition of the AJCC staging manual was used for tumour classification [31].

### 4.3. Immunohistochemistry and Immunofluorescence

MVD was assessed in 43 cases with IHC for CD34 as described previously [3]. Counts were represented as vessels/mm^2^. Numbers of lymphocytes and macrophages were assessed as described previously [2,19]. T cells were detected with IF using antibodies against CD3, CD4, CD8 and FoxP3. Counts were represented as number of cells/mm^2^. Macrophages were detected with IF using antibodies against CD68, CD163, and CD68CD163 double-staining. Counts were determined in pixels/mm^2^. BAP1 status was assessed with IHC as described previously [32]. Nuclear BAP1 staining was scored by an experienced ocular pathologist, and categorized as BAP1-positive or BAP1-negative. Extravascular networks were identified with PAS staining; closed vascular structures were named “loops”, and at least 3 adjacent loops were named “networks”.

### 4.4. Chromosome 3/8q Status and Gene Expression

The QIAmp DNA Mini Kit was used to isolate DNA for single nucleotide polymorphism (SNP) analysis according to guidelines of the manufacturer (Qiagen, Venlo, The Netherlands). Status of chromosome 3 was determined with SNP analysis performed with the Affymetrix 250K_NSP chip and the Affymetrix Cytoscan HD chip (Affymetrix, Santa Clara, CA, USA) [14]. The copy number of chromosome 8q was identified with ddPCR. A threshold of >2.1 was defined as gain of 8q [14]. The RNeasy Mini Kit was used to isolate mRNA for gene expression analysis (Qiagen, Venlo, The Netherlands). Gene expression levels were obtained using the Illumina HT-12 v4 chip (Illumina, San Diego, CA, USA). Angiogenesis-related factors were selected based on literature regarding angiogenesis. Only these predefined genes were assessed in the current analysis (Table S5).

### 4.5. TCGA Data

Findings were corroborated using mRNA data from 80 UM patients from the TCGA project: http://cancergenome.nih.gov/ [33]. In this set, BAP1 expression was provided as mRNA expression levels, and dichotomized into BAP1-positive and BAP1-negative tumours, using the median [13].

### 4.6. Statistical Analysis

Analyses were performed using SPSS version 23 (I.B.M.). Categorical data was analysed with Chi-square tests. Numerical data was analysed with the Mann–Whitney *U* test between 2 groups, and with the Jonckheere test between multiple groups with a trend. Correlations were assessed with the Spearman’s test. Linear regression was performed for univariate and multivariate analyses. Survival data was analysed with the Kaplan–Meier method and log-rank tests; groups of high and low MVD and mRNA gene expression were based on the median. *p*-values < 0.05 were considered statistically significant.

## 5. Conclusions

In conclusion, we demonstrated that the genetic evolution of UM not only involves tumour infiltrate, but also tumour angiogenesis. Late events (such as BAP1 loss) are related to an increase in MVD, while early events (such as 8q gain) are not. Gain of 8q may be related to a preparation phase; however, as several angiogenesis-related genes are already expressed differentially in the absence of monosomy 3/BAP1 loss. We observed new associations with MVD, such as with monosomy 3/BAP1 loss, an increased count of lymphocytes, and a decreased expression of VEGF-B, indicating that more (and other) mechanisms are involved in angiogenesis of UM than previously thought.

## Figures and Tables

**Table 1:** Patient and tumour characteristics of 43 uveal melanoma patients for whom data on micro-vascular density (MVD) were available.

Categorical	Total	MVD	*p*-Value
	Cases (%)	Median	
Gender			
Male	23 (53)	89.0	0.85 ^#^
Female	20 (47)	88.5	
Side			
OD	23 (53)	86.0	0.95 ^#^
OS	20 (47)	93.5	
TNM stage (8th)			
T1	4 (9)	79.0	0.23 *
T2	14 (33)	82.0	
T3	25 (58)	91.0	
Pigmentation			
Light	29 (67)	89.0	0.97 ^#^
Dark	14 (33)	96.5	
Cell Type			
Spindle	11 (26)	69.0	0.009 ^#^
Mixed + Epithelioid	32 (74)	100.0	
Ciliary Body Involvement			
No	24 (56)	89.0	0.58 ^#^
Yes	19 (44)	103.0	
Loops and Networks			
None	7 (16)	71.0	0.052 *
Loops+, networks−	8 (19)	83.0	
Loops+, networks+	27 (63)	102.0	
Metastasis			
No	25 (58)	76.0	0.010 ^#^
Yes	18 (42)	110.0	
Melanoma-Related Death			
No	25 (58)	76.0	0.010 ^#^
Yes	18 (42)	110.0	
**NUMERICAL**	**Total**	**Correlation**	***p*-Value**
		**Spearman**	
Age—Median	63.6	0.135	0.390
LBD—Median	13.0	0.299	0.051
Prominence—Median	8.0	−0.278	0.072

*p* values were calculated with: ^#^ Mann–Whitney *U* test, * Jonckheere test for trend. (Abbreviations: TNM, tumour-node-metastasis; LBD, largest basal diameter).

**Table 2:** mRNA expression of angiogenesis-related genes in relation to MVD (*n* = 28).

mRNA	Median (Range)	Spearman Corr.	*p*-Value
VEGF-A	6.76 (6.51–7.34)	0.005	0.989
VEGF-B	8.54 (7.6–9.41)	−0.425	0.024 *
VEGF-C	6.73 (6.37–7.73)	0.209	0.286
HIF1A	7.21 (6.89–7.91)	0.327	0.089
VHL	7.96 (7.35–8.54)	−0.226	0.248
ANGPT1	6.57 (6.31–7.04)	0.155	0.431
ANGPT2	6.54 (6.23–8.19)	0.389	0.041 *
PDGFA	6.96 (6.46–7.82)	0.060	0.761
CD34	7.37 (6.73–7.9)	0.497	0.007 *
CDH1	10.74 (5.8–12.94)	0.337	0.079
PECAM1	7.23 (6.68–9.57)	0.367	0.055
VWF	9.86 (8.62–11.14)	0.479	0.010 *

* *p*-value < 0.05.

## Supplementary Materials

The following are available online at https://www.mdpi.com/2072-6694/11/7/979/s1, Table S1: Patient and tumour characteristics of UM patients with data on MVD and mRNA expression, Table S2: Patient and tumour characteristics of UM patients with data on tumour genetics and mRNA expression, Table S3: mRNA expression of angiogenesis-related genes (Leiden data), Table S4: mRNA expression of angiogenesis-related genes in (TCGA data), Table S5: Overview of angiogenesis-related genes.
