# Supplementary material for: Tumour Angiogenesis in Uveal Melanoma Is Related to Genetic Evolution
Source: Cancers (Basel). 2019 Jul 13;11(7):979. doi: 10.3390/cancers11070979 (PMC6678109; doi:10.3390/cancers11070979)
Supplement: Supplementary file 1 [file cancers-11-00979-s001.zip › Supplemental Table S5.pdf]

Supplemental Table S5. Overview of angiogenesis-related genes.

mRNA	Name	Locus	EntrezID	Probe name	Probe number	Remarks
VEGF-A	VEGF-A	6p21.1	7422	VEGFA_p1	ILMN_2375879	
VEGF-B	VEGF-B	11q13.1	7423	VEGFB_p3	ILMN_1722855	
VEGF-C	VEGF-C	4q34.3	7424	VEGFC_p1	ILMN_1701204	
HIF1A	Hypoxia Inducible Factor-1alfa	14q23.2	3091	HIF1A_p1	ILMN_2379788	
VHL	Von Hippel Lindau	3p25.3	7428	VHL_p2	ILMN_1738579	
ANGPT1	Angiopoietin 1	8q23.1	284	ANGPT1_p1	ILMN_1677723	
ANGPT2	Angiopoietin 2	8q23.1	285	ANGPT2_p2	ILMN_1774207	
PDGFA	Platelet-derived Growth Factor-A	7p22.3	5154	PDGFA_p1	ILMN_2342695	
CD34	CD34	1q32.2	947	CD34_p1	ILMN_2341229	
CDH1	Cadherin-1	16q22.1	999	CDH1_p1	ILMN_1770940	Also known as: epithelial cadherin (e-cadherin)
PECAM1	Platelet Endothelial Cell Adhesion Molecule 1	17q23.3	5175	PECAM1_p1	ILMN_1689518	Also known as: CD31
VWF	Von Willebrand Factor	12p13.31	7450	VWF_p1	ILMN_1752755	
CD3D	CD3	11q23.3	915	CD3D_p1	ILMN_2261416	Lymphocyte marker
CD4	CD4	12p13.31	920	CD4_p1	ILMN_1727284	Lymphocyte marker
CD8A	CD8	2p11.2	925	CD8A_p3	ILMN_2353732	Lymphocyte marker
CD68	CD68	17p13.1	968	CD68_p1	ILMN_1714861	Macrophage marker
CD163	CD163	12p13.31	9332	CD163_p2	ILMN_2379599	Macrophage marker
BAP1	BRCA1 Associated Protein1	3p21.1	8314	BAP1_p1	ILMN_1768363	
