# Supplementary material for: Tumour Angiogenesis in Uveal Melanoma Is Related to Genetic Evolution
Source: Cancers (Basel). 2019 Jul 13;11(7):979. doi: 10.3390/cancers11070979 (PMC6678109; doi:10.3390/cancers11070979)
Supplement: Supplementary file 1 [file cancers-11-00979-s001.zip › Supplemental Table S1.pdf]

Supplemental Table S1. Patient and tumour characteristics of UM patients with data on MVD and mRNA expression (n = 28).

**CATEGORICAL**	**Total Cases (%)**
Gender	
Male	15 (54)
Female	13 (46)
Side	
OD	14 (50)
OS	14 (50)
TNM stage (8th)	
T1	4 (14)
T2	9 (32)
T3	15 (54)
Pigmentation	
Light	22 (79)
Dark	6 (21)
Cell Type	
Spindle	10 (36)
Mixed + Epithelioid	18 (64)
Ciliary body involvement	
No	16 (57)
Yes	12 (43)
Loops and Networks	
None	7 (25)
Loops+, networks-	5 (18)
Loops+, networks+	16 (57)
Metastasis	
No	14 (50)
Yes	14 (50)
Melanoma-Related Death	
No	14 (50)
Yes	14 (50)

**NUMERICAL**	
Age – Median	68.9
LBD – Median	14.5
Prominence - Median	7.0
