# Supplementary material for: Tumour Angiogenesis in Uveal Melanoma Is Related to Genetic Evolution
Source: Cancers (Basel). 2019 Jul 13;11(7):979. doi: 10.3390/cancers11070979 (PMC6678109; doi:10.3390/cancers11070979)
Supplement: Supplementary file 1 [file cancers-11-00979-s001.zip › Supplemental Table S3.pdf]

Supplemental Table S3. mRNA expression of angiogenesis-related genes in relation to 8q gain and BAP1 loss in 24 and 54 cases, respectively (Leiden data).

mRNA		BAP1+ 8q normal *n* = 11 Median	BAP1+ 8q gain *n* = 13 Median	*p*			BAP1+ *n* = 24 Median	BAP1- *n* = 30 Median	*p*	
VEGF-A		6.75	6.81	0.125			6.80	6.76	0.651	
VEGF-B		8.83	8.66	0.022*	↓		8.74	8.44	<0.001*	↓
VEGF-C		6.86	6.62	0.026*	↓		6.76	6.68	0.651	
HIF1A		6.99	7.10	0.087			7.02	7.28	<0.001*	↑
VHL		8.22	8.15	0.284			8.17	7.71	0.003*	↓
ANGPT1		6.58	6.63	0.931			6.59	6.55	0.508	
ANGPT2		6.42	6.52	0.040*	↑		6.45	6.58	0.015*	↑
PDGFA		6.94	6.97	0.839			6.96	6.91	0.384	
CD34		6.94	7.24	0.401			7.22	7.37	0.126	
CDH1		9.57	9.82	0.235			9.73	11.58	<0.001*	↑
PECAM1		6.80	6.99	0.140			6.95	7.28	0.004*	↑
VWF		9.47	9.83	0.839			9.57	9.97	0.013*	↑
CD3D		6.46	6.67	0.026*	↑		6.59	7.18	0.015*	↑
CD4		6.41	6.58	0.077			6.53	6.66	0.042*	↑
CD8A		6.55	6.72	0.125			6.63	7.50	0.016*	↑
CD68		9.76	10.86	0.007*	↑		10.24	11.23	0.002*	↑
CD163		6.82	7.31	0.125			7.01	7.19	0.394	
BAP1		7.96	7.95	0.977			7.96	7.26	<0.001*	↓

**p*-value <0.05. (Abbreviations: BAP1+, BAP1-positive; BAP1-, BAP1-negative; 8q normal, normal chromosome 8q; 8q gain, gain of chromosome 8q)
