# Supplementary material for: Tumour Angiogenesis in Uveal Melanoma Is Related to Genetic Evolution
Source: Cancers (Basel). 2019 Jul 13;11(7):979. doi: 10.3390/cancers11070979 (PMC6678109; doi:10.3390/cancers11070979)
Supplement: Supplementary file 1 [file cancers-11-00979-s001.zip › Supplemental Table S4.pdf]

Supplemental Table S4. mRNA expression of angiogenesis-related genes in relation to 8q gain or BAP1 loss in 40 and 40 patients, respectively (TCGA data).

		BAP1+ 8q normal	BAP1+ 8q gain				BAP1+	BAP1-		
mRNA		n = 17 Median	n = 23 Median	p			n = 40 Median	n = 40 Median	p	
VEGF-A		7.67	8.23	0.151			8.12	8.64	0.001*	↑
VEGF-B		12.90	13.07	0.503			12.96	12.17	<0.001*	↓
VEGF-C		6.49	5.27	0.069			5.36	6.24	0.059	
HIF1A		10.05	9.21	0.061			9.69	10.27	0.002*	↑
VHL		8.49	7.95	0.176			8.27	7.91	0.023*	↓
ANGPT1		1.89	1.76	0.519			1.76	2.69	0.022*	↑
ANGPT2		4.33	4.75	0.159			4.62	5.62	<0.001*	↑
PDGFA		9.97	9.08	0.002*	↓		9.43	9.83	0.006*	↑
CD34		8.09	8.09	0.753			8.09	8.10	0.665	
CDH1		12.43	12.41	0.880			12.42	14.06	<0.001*	↑
PECAM1		7.27	7.44	0.712			7.35	7.95	<0.001*	↑
VWF		11.10	11.23	0.753			11.16	10.95	0.651	
CD3D		1.37	2.33	0.924			1.88	4.18	<0.001*	↑
CD4		8.76	9.24	0.082			9.02	9.19	0.810	
CD8A		3.87	3.02	0.359			3.37	6.23	<0.001*	↑
CD68		10.95	12.07	<0.001*	↑		11.68	11.98	0.149	
CD163		7.50	7.02	0.159			7.14	7.69	0.149	
BAP1		11.97	12.28	0.136			12.09	9.71	<0.001*	↓

*p-value <0.05. (Abbreviations: BAP1+, BAP1-positive; BAP1-, BAP1-negative; 8q normal, normal chromosome 8q; 8q gain, gain of chromosome 8q)
