# Supplementary material for: Tumour Angiogenesis in Uveal Melanoma Is Related to Genetic Evolution
Source: Cancers (Basel). 2019 Jul 13;11(7):979. doi: 10.3390/cancers11070979 (PMC6678109; doi:10.3390/cancers11070979)
Supplement: Supplementary file 1 [file cancers-11-00979-s001.zip › Supplemental Table S2.pdf]

Supplemental Table S2. Patient and tumour characteristics of UM patients with data on tumour genetics and mRNA expression (n = 54).

CATEGORICAL	Total Cases (%)
Gender	
Male	28 (52)
Female	26 (48)
Side	
OD	26 (48)
OS	28 (52)
TNM stage (8th)	
T1	2 (4)
T2	23 (43)
T3	27 (50)
T4	2 (4)
Pigmentation*	
Light	36 (67)
Dark	17 (32)
Cell Type	
Spindle	19 (35)
Mixed + Epithelioid	35 (65)
Ciliary body involvement*	
No	32 (59)
Yes	21 (39)
Loops and Networks*	
None	16 (30)
Loops+, networks-	10 (19)
Loops+, networks+	27 (50)
Metastasis	
No	23 (43)
Yes	31 (57)
Melanoma-Related Death	
No	23 (43)
Yes	31 (57)

NUMERICAL	Total
Age – Median	64.0
LBD – Median	14.0
Prominence - Median	8.0

*Rows do not add up to 100% due to one missing value.
